# Inverse and reciprocal regulation of p53/p21 and Bmi-1 modulates vasculogenic differentiation of dental pulp stem cells

**DOI:** 10.1038/s41419-021-03925-z

**Published:** 2021-06-24

**Authors:** Zhaocheng Zhang, Min Oh, Jun-Ichi Sasaki, Jacques E. Nör

**Affiliations:** 1 Angiogenesis Research Laboratory, Department of Cariology, Restorative Sciences and Endodontics, University of Michigan School of Dentistry, Ann Arbor, MI 48109 USA; 2 Department of Biomedical Engineering, University of Michigan College of Engineering, Ann Arbor, MI USA; 3 Department of Otolaryngology, University of Michigan School of Medicine, Ann Arbor, MI USA

**Keywords:** Morphogen signalling, Mesenchymal stem cells, Stem-cell differentiation

## Abstract

Dental pulp stem cells (DPSC) are capable of differentiating into vascular endothelial cells. Although the capacity of vascular endothelial growth factor (VEGF) to induce endothelial differentiation of stem cells is well established, mechanisms that maintain stemness and prevent vasculogenic differentiation remain unclear. Here, we tested the hypothesis that p53 signaling through p21 and Bmi-1 maintains stemness and inhibits vasculogenic differentiation. To address this hypothesis, we used primary human DPSC from permanent teeth and Stem cells from Human Exfoliated Deciduous (SHED) teeth as models of postnatal mesenchymal stem cells. DPSC seeded in biodegradable scaffolds and transplanted into immunodeficient mice generated mature human blood vessels invested with smooth muscle actin-positive mural cells. Knockdown of p53 was sufficient to induce vasculogenic differentiation of DPSC (without vasculogenic differentiation medium containing VEGF), as shown by increased expression of endothelial markers (VEGFR2, Tie-2, CD31, VE-cadherin), increased capillary sprouting in vitro; and increased DPSC-derived blood vessel density in vivo. Conversely, induction of p53 expression with small molecule inhibitors of the p53-MDM2 binding (MI-773, APG-115) was sufficient to inhibit VEGF-induced vasculogenic differentiation. Considering that p21 is a major downstream effector of p53, we knocked down p21 in DPSC and observed an increase in capillary sprouting that mimicked results observed when p53 was knocked down. Stabilization of ubiquitin activity was sufficient to induce p53 and p21 expression and reduce capillary sprouting. Interestingly, we observed an inverse and reciprocal correlation between p53/p21 and the expression of Bmi-1, a major regulator of stem cell self-renewal. Further, direct inhibition of Bmi-1 with PTC-209 resulted in blockade of capillary-like sprout formation. Collectively, these data demonstrate that p53/p21 functions through Bmi-1 to prevent the vasculogenic differentiation of DPSC.

## Introduction

Mesenchymal stem cells derived from the cranial neural crest are essential for tooth development. They generate most of the soft and hard tissues in and around the tooth, including dental pulp, dentin, periodontal ligament, and alveolar bone [1–3]. Stem cells found in the dental pulp are embryologically derived from the neural crest and were named dental pulp stem cells (DPSC) in permanent teeth [4] and Stem cells from Human Exfoliated Deciduous teeth (SHED) in primary teeth [5]. Both, DPSC and SHED exhibit the capacity of differentiating into several cell types (i.e., multipotency) including osteoblasts, chondrocytes, adipocytes, odontoblasts, smooth muscle cells, neuronal cells [5–8], and self-renewal [9, 10]. We have shown that DPSCs can also differentiate into endothelial cells that generate functional blood vessels when transplanted into murine hosts [11, 12]. Understanding the mechanisms that define decisions regulating the vasculogenic fate of MSCs will help us understand processes underlying physiological tissue regeneration and repair. It will also allow us to use these cells in regenerative medicine as long as one uses approaches that enable vasculogenic differentiation in absence of animal serum, as shown [13, 14].

We observed that blood vessel generated with stem cells of dental origin mature and become invested by mural cells, e.g., pericytes or vascular smooth muscle cells (VSMC). Pericytes surround blood vessels and regulate numerous functions, such as vessel growth, stabilization, and permeability [15, 16]. Pericytes distinguish from VSMC by their location relative to the endothelium, their morphology, and their cell marker expression. Several markers have been used to identify pericytes, including smooth muscle actin (SMA), desmin, NG-2, platelet-derived growth factor receptor (PDGFR)-β, CD146, and NG-2 [17, 18]. Pericytes also express surface molecules commonly used to identify MSCs, such as CD44, CD73, CD90, CD105 [18]. Notably, the expression pattern of these markers is fluid and indicates the stemness/differentiation state of these cells in response to microenvironmental cues that trigger intracellular events that define cell fate.

p53 is a key tumor suppressor that protects cells from transformation by inducing cell cycle arrest and/or apoptosis [19]. Emerging evidence suggests that p53 also has a major role in embryonic development by modulating cell differentiation. Indeed, increased p53 activity is incompatible with normal embryogenesis. As such, the inactivation of physiological inhibitors of p53 function (e.g., MDM2) causes embryonic lethality that can be rescued by the deletion of p53 [20]. Specific examples of p53′s function in development include (A) inhibition of p53 upon activation of β-catenin signaling is essential for artery formation during embryogenesis [21]; (B) muscle differentiation in response to genotoxic stress is suppressed by p53 [22]; and (C) inactivation of p53 in human keratinocytes leads to squamous differentiation [23]. However, the role of p53 in vasculogenic differentiation of postnatal mesenchymal stem cells remains unclear.

Bmi-1 is a polycomb protein that has a key role in the regulation of embryonic stem cell differentiation and is essential for the self-renewal of adult hematopoietic stem cells [24]. Interestingly, Bmi-1 inhibits Hox genes to maintain adult stem cells and to prevent deregulated differentiation of dental stem cells in mouse models [25]. A link between the p53/p21 pathway and Bmi-1 has been established in neural stem cells (NSCs). Although knockdown of Bmi-1 inhibits NSC self-renewal, concomitant silencing of p21 rescues Bmi-1-knockdown-mediated inhibition of self-renewal and neurosphere formation [26]. As such, it is plausible that the crosstalk between p53/p21 and Bmi-1 has a role in the regulation of the fate of dental stem cells.

Although emerging data suggest that p53/p21 regulates stem cell fate, the impact of this signaling pathway on the vasculogenic differentiation of mesenchymal stem cells is unknown. Here, we observed that p53 inhibition is sufficient to drive the vasculogenic differentiation of dental pulp stem cells, whereas p53 induction blocks this effect. We also showed an inverse correlation between p53/p21 and Bmi-1 that defines the vasculogenic fate of these stem cells. Collectively, this work unveils a key role for p53/p21 signaling through Bmi-1 in the regulation of the vasculogenic differentiation of mesenchymal stem cells of dental origin.

## Results

### p53 represses vasculogenic differentiation of DPSC

**Fig. 1:**
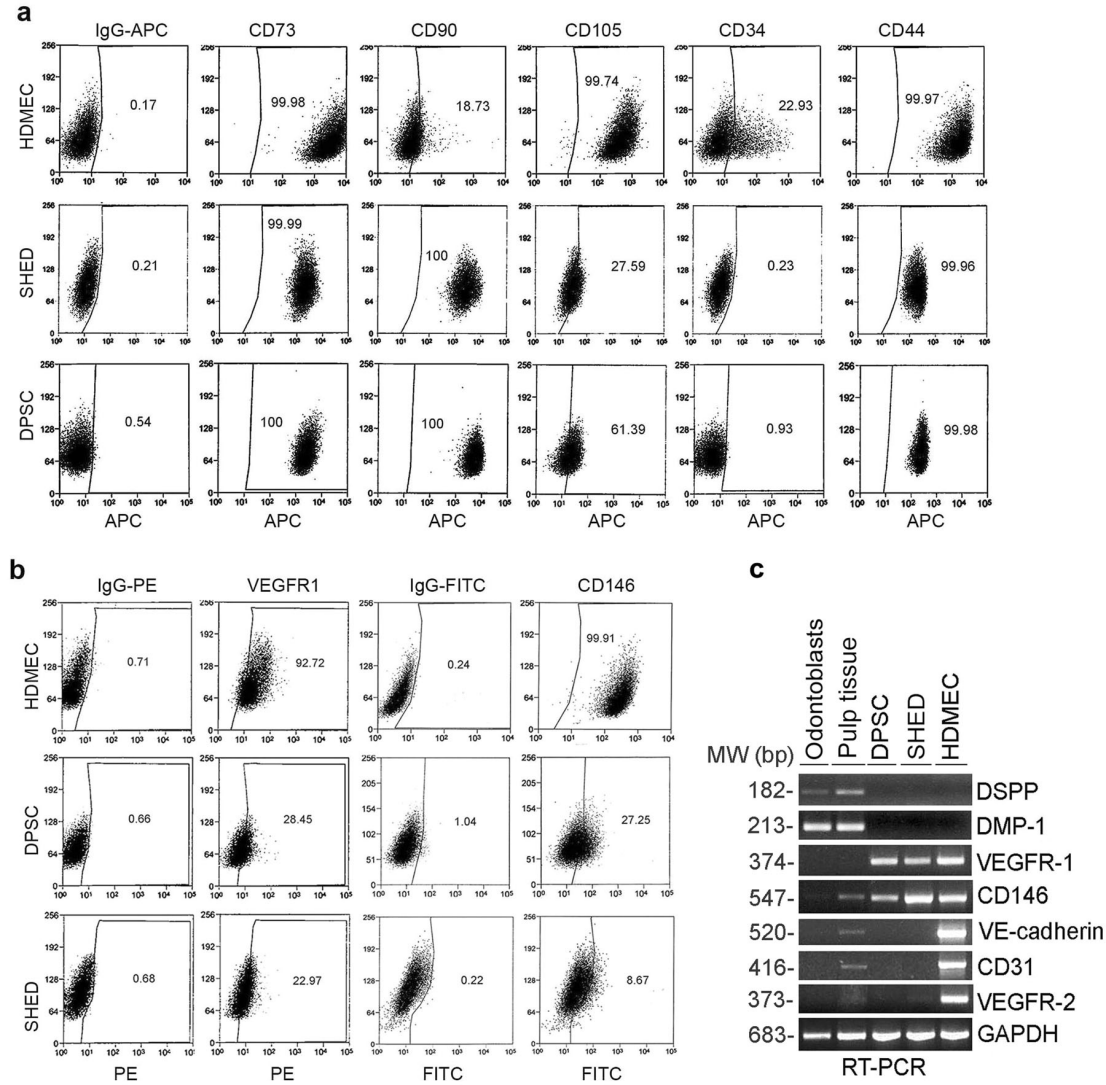
Characterization of MSC and endothelial markers in dental pulp stem cells. Cell surface markers detected by flow cytometry in DPSC, SHED, and HDMEC under standard culture conditions for each cell type. **a** Flow plots depicting expression of MSC markers (i.e., CD73, CD90, CD105, CD34, CD44) using IgG-APC as isotype-matched control. **b** Flow plots depicting expression of endothelial cell-related proteins (VEGFR1 and CD146) using IgG-PE and IgG-FITC were used as isotype-matched controls. **c** RT-PCR for odontogenic/osteogenic markers (DSPP, DMP-1), endothelial cell-related markers (VEGFR1, VEGFR2, CD146, CD31, VE-cadherin) in primary human odontoblasts, dental pulp tissue, DPSC, SHED, or HDMEC. GAPDH was used as a loading control.

To characterize the primary human DPSC used here, we performed flow cytometry analyses of primary human DPSC and SHED for expression of mesenchymal stem cell and vasculogenic markers using primary human dermal microvascular endothelial cells (HDMEC) as controls. Both pulp stem cell types expressed mesenchymal stem cell markers (e.g., CD73, CD90, CD105), and did not express the negative marker CD34 (Fig. 1a), which is consistent with the consensus from the Mesenchymal and Tissue Stem Cell Committee of the International Society for Cellular Therapy [27]. As expected, while all pulp stem cells expressing CD90 [28], <20% of the HDMEC expressed this marker. In contrast, although the majority of the HDMEC cells expressed CD105 (Endoglin) [29], CD146 [30], and vascular endothelial growth factor (VEGF)R1 [11, 31], a smaller fraction of DPSC expressed these markers (Fig. 1a, b). The specificity of expression of vasculogenic markers at the RNA level was verified by reverse transcription polymerase chain reaction (RT-PCR) (Fig. 1c). Although unstimulated DPSC and SHED expressed VEGFR1 and CD146 at baseline, they did not express VEGFR2, CD31, and VE-cadherin, as we have previously shown [12]. As controls for this experiment, we used primary human odontoblasts scrapped from the pre-dentin of extracted teeth that showed expression of the odontoblastic/osteoblastic markers DSPP and DMP-1 (Fig. 1c). We also prepared total RNA from human dental pulp tissue that showed expression of DSPP and DMP-1, and low-level expression of the vasculogenic markers (Fig. 1c).

**Fig. 2:**
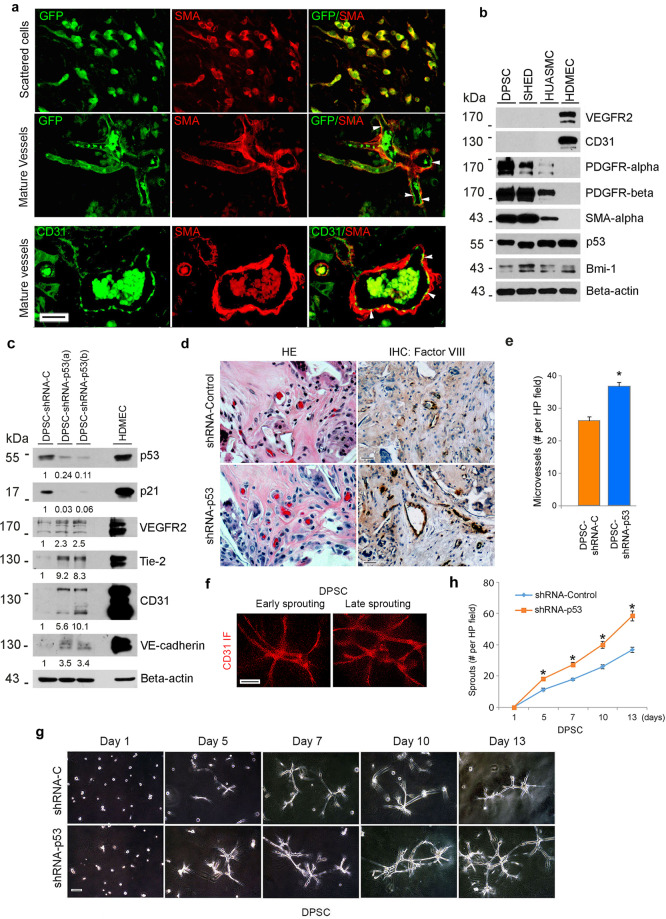
Silencing p53 enhances vasculogenic differentiation of DPSC in vitro and in vivo. **a** Photomicrographs depicting dental pulp stem cells that differentiated into functional blood vessels in vivo. GFP-transduced DPSC were seeded in scaffolds and transplanted in the subcutaneous space of the dorsum of SCID mice. Six weeks later, scaffolds were retrieved and tissue sections were prepared for immunofluorescence staining. Scattered cells showed positive staining for both, GFP (green), and SMA-alpha (red). In mature blood vessels, GFP was detected in the endothelial cells, SMA-alpha was stained the surrounding smooth muscle cells. Endothelial cells in the inner layer were stained for CD31 (green), while surrounding smooth muscle cells stained positive for SMA-alpha (red). Arrows point to DPSC-derived endothelial cells (green cells). Scale bar: 50 µm. **b** Western blots for VEGFR2, CD31, PDGFR-a, PDGFR-β, SMA-a, p53, and Bmi-1 in DPSC, SHED, HUASMC, and HDMEC. **c** Endothelial cell differentiation: shRNA-transduced DPSC were cultured with 5%FBS–MEM for 14 days, western blots for p53, p21, VEGFR2, Tie-2, CD31, and VE-cadherin. The density of protein expression was normalized with β-actin. **d** shRNA-p53-mediated vasculogenic differentiation in vivo: H&E and IHC staining. Blood vessels were revealed by H&E staining and detected with anti-factor VIII antibody, scale bar: 50 µm. **e** Graph depicting the blood vessel density observed in **d** stained with Factor VIII. Asterisk indicates *p* = 0.00035, as determined by *t* test. **f** DPSC cells were seeded in matrigel and cultured with EGM2 for 8 days. The Matrigel was fixed, and the sprouts were revealed by IF staining for CD31. Scale bar: 100 µm. **g** In all, 1 × 10^4^ shRNA-transduced DPSC were seeded in growth factor-reduced matrigel-coated 12-well plate and cultured in endothelial differentiation medium (EGM2) for indicated time points. Sprouts were photographed, scale bar:100 µm. **h** Graph depicting the numbers of sprout formed in **g**. Three independent experiments using triplicate wells per condition were performed. Asterisk indicates *p* < 0.001, as determined by one-way ANOVA followed by a post hoc test (Tukey’s test).

To verify the vasculogenic differentiation potential of DPSC in vivo, green fluorescence protein (GFP)-transduced DPSC were transplanted into immunodeficient mice. Early stage, DPSC-GFP cells were scattered throughout the scaffold and expressed SMA-alpha (Fig. 2a). As these DPSC-GFP cells differentiated into functional mature CD31-positive blood vessels, the DPSC-GFP cells lost SMA-alpha expression and recruited mural (SMA-alpha positive) cells from the host (Fig. 2a). This differentiation process resulted in blood-containing blood vessels lined with human DPSC-derived (i.e., GFP-positive) endothelial cells (i.e., CD31-positive) lined with host (i.e., GFP-negative) mural cells (i.e., SMA-alpha-positive) (Fig. 2a). Notably, unstimulated DPSC and SHED expressed markers of perivascular mural cells (i.e., PDGFR-a, PDGFR-β and SMA-alpha) using human umbilical artery smooth muscle cells (HUASMC) as controls, but did not express markers of endothelial cells (i.e., VEGFR2, CD31) using HDMEC as controls in vitro (Fig. 2b). Together, these data indicated that DPSC exhibit features of perivascular cells while unstimulated but have the capacity to switch to a vascular endothelial phenotype when transplanted in murine hosts.

While performing the characterization of the DPSC, we observed that they consistently express high levels of the tumor suppressor p53 (Fig. 2b), which has also been implicated in the regulation of cell fate during physiological development [32]. To begin to understand the role of p53 on the vasculogenic differentiation of DPSC, we stably transduced shRNA constructs to silence expression of p53 in these cells, or scrambled sequence vector controls (Fig. 2c). We observed that knockdown of p53 was sufficient to downregulate p21 and induce expression of endothelial markers (i.e., VEGFR2, Tie-2, CD31, and VE-cadherin) in DPSC grown in standard cell culture medium (i.e., without rhVEGF_165_ supplementation), indicating that p53 serves as a “gate-keeper” for vasculogenic differentiation of these cells (Fig. 2c). We then seeded these cells in biodegradable scaffolds and transplanted them in mice and observed an increase in the density of human blood vessels in scaffolds seeded with p53-silenced DPSC cells (*p* < 0.05), when compared with scaffolds seeded with vector control cells (Fig. 2d, e). Human blood vessels were identified with human specific anti-CD31 antibody, as we showed [33]. To confirm the vascular phenotype of the capillary-like structures derived from DPSC in matrigel, we performed direct immunofluorescence and observed that the DPSC-derived sprouts are positive for CD31, a classical marker of mature endothelial cells (Fig. 2f). The increase in the vasculogenic potential of p53-silenced DPSC cells was observed by using the in vitro capillary-tube assay. Knockdown of p53 was sufficient to increase the number of capillary-like sprouts generated by DPSC cells seeded in matrigel (*p* < 0.05), when compared to vector controls (Fig. 2g, h).

**Fig. 3:**
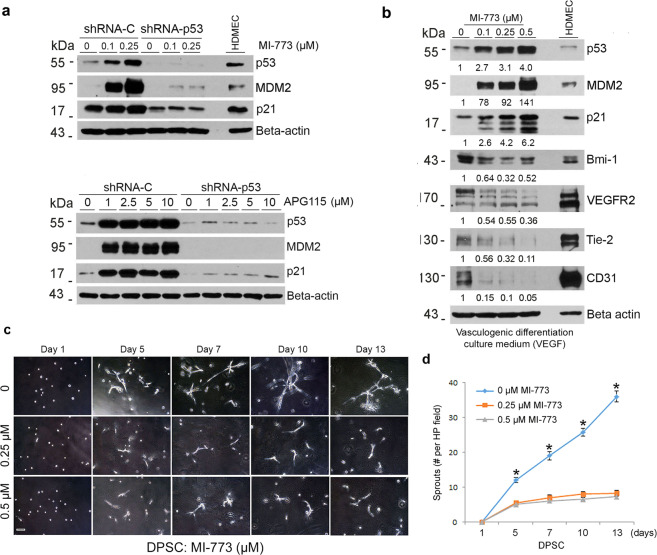
Stabilization of p53 by blockade of the p53-MDM2 binding inhibits vasculogenic differentiation of dental pulp stem cells. **a** shRNA-p53 transduced DPSC were treated with 0–2.5 µM MI-773 and 0–10 µM APG-115 for 24 h, western blots were performed for P53, MDM2, and p21, β-actin was used as loading control. **b** DPSC were cultured with endothelial differentiation medium in the presence of 50 ng/ml VEGF and 0–0.25 µM MI-773 for 14 days, western blots for p53, MDM2, p21, Bmi-1, VEGFR2, Tie-2, and CD31. The density of protein expression was normalized with β-actin. **c**, **d** 1 × 10^4^ DPSC were seeded in growth factor-reduced matrigel and cultured in endothelial differentiation medium (EGM2) in the presence of 0–0.5 µM MI-773 for 13 days. **c** photographs of sprout, scale bar:100 µm. **d** Graph depicting the number of sprouts formed in **c**. Asterisk indicates *p* < 0.001, as determined by one-way ANOVA followed by a post hoc test (Tukey’s test).

To verify these intriguing results, we performed the reverse experiment using two different small molecule inhibitors of the p53-MDM2 interaction (MI-773 or APG-115) that are known to stabilize p53 and therefore increase the expression levels of p53 and downstream p21, as well as MDM2 via a feedback loop [34, 35]. As expected, we observed that MI-773 (as well as APG-115) induced dose-dependent expression of p53, MDM2, and p21 in shRNA vector control DPSC cells, but not in p53-silenced DPSC cells (Fig. 3a). Then, we exposed DPSC cells to a vasculogenic differentiation medium (containing rhVEGF_165_) in presence of increasing concentrations of the small molecule inhibitor of p53-MDM2 interaction (Fig. 3b). Induction of p53 signaling with MI-773 was sufficient to inhibit vasculogenic differentiation as determined by decreased expression of endothelial markers (i.e., VEGFR2, Tie-2, CD31) (Fig. 3b) and inhibition of in vitro capillary sprouting (Fig. 3c, d). The decrease in capillary sprouting observed upon MI-773 treatment in DPSC cells was verified using SHED cells (Supplementary Fig. S1). Surprisingly, we also observed a dose-dependent inhibition of Bmi-1, a major regulator of self-renewal (Fig. 3b), which will be explored in more detail in the experiments described below.

### p21 is a downstream effector of p53-mediated regulation of the vasculogenic fate of DPSC

**Fig. 4:**
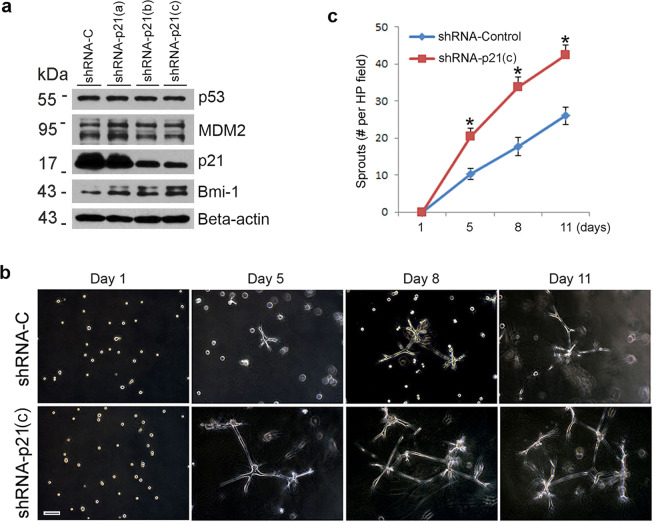
p21 is a downstream effector of p53-mediated regulation of the vasculogenic fate of dental pulp stem cells. **a** DPSC were transduced with shRNA scramble and shRNA-p21, western blot was performed for p53, MDM2, and Bmi-1. **b**, **c** shRNA scramble and shRNA-p21 transduced DPSC were seeded in growth factor-reduced matrigel and cultured in endothelial differentiation medium for different time points. **b** photographs of sprouts, scale bar:100 µm. **c** Graph depicting the number of sprouts formed in **b**. Asterisk indicates *p* < 0.001, as determined by one-way ANOVA followed by a post hoc test (Tukey’s test).

To understand the series of events involved in the regulation of the vasculogenic fate by p53, we bypassed p53 and silenced expression of its downstream effector p21 (Fig. 4a). Of the three shRNA-p21 constructs that we used, construct (c) was the most efficient and was used for the remaining experiments included here. Similar to what we observed upon silencing of p53, knocking down p21 was sufficient to increase the number of capillary-like sprouts generated by DPSC cells cultured in 3D matrigel plates (*p* < 0.05) when compared with scrambled sequence vector control cells (Fig. 4b, c). Considering that silencing p21 had no impact on p53 or MDM2 expression (Fig. 4a), one concludes that p21 functions as a downstream effector of p53 in the regulation of the vasculogenic fate of DPSC. Consistent with the data described above for p53, p21 downregulation is accompanied by a reciprocal upregulation of Bmi-1 (Fig. 4a).

### p53-dependent vasculogenic differentiation requires inverse and reciprocal regulation of p21 and Bmi-1

**Fig. 5:**
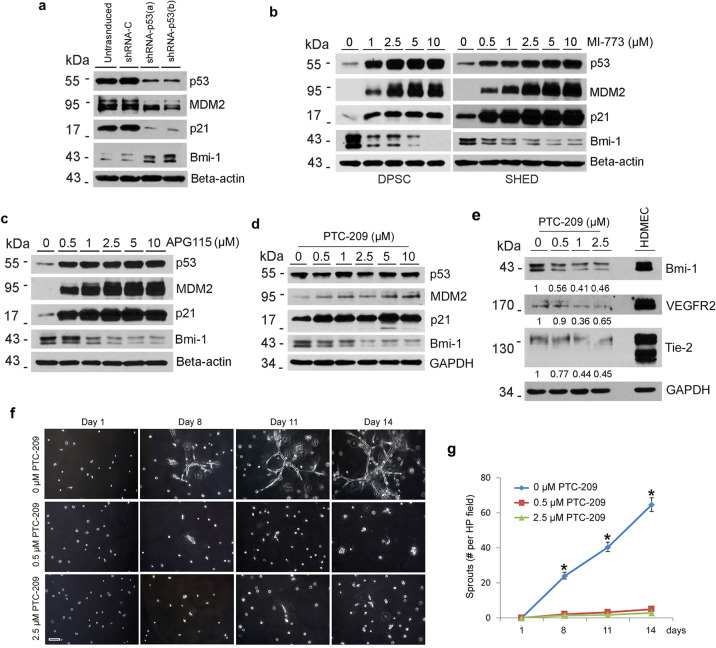
p53-dependent vasculogenic differentiation requires inverse and reciprocal regulation of p21 and Bmi-1. **a**–**d** Western blot for p53, MDM2, p21, and Bmi-1 in dental pulp cells. **a** Untransduced and shRNA-transduced DPSC. **b**–**d** Dental pulp cells were treated with 0–10 µM MI-773 (**b**), 0–10 µM APG-115 (**c**), and 0–10 µM Bmi-1 inhibitor (PTC-209) (**d**) for 24 h. **e** SHED were cultured with 5% FBS–MEM in the presence of 50 ng/ml VEGF with or without 0–2.5 µM PTC-209 for 14 days, western blots for VEGFR2, Tie-2, Bmi-1, and p21, GAPDH was used as a loading control. The density of protein expression was normalized with GAPDH. **f**, **g** 1 × 10^4^ SHED were seeded in growth factor-reduced matrigel and cultured with EGM2 in the presence of 0–2.5 µM PTC-209 for different time points. **f** photographs of sprout in SHED. Scale bar: 100 µm. **g** Graph depicting the number of sprouts formed in **f**, Asterisk indicates *p* < 0.001, as determined by one-way ANOVA followed by a post hoc test (Tukey’s test).

p21-dependent Bmi-1 regulation has an important role in NSCs self-renewal during development [26]. To begin to understand the relationship between p53/p21 and downstream Bmi-1, we used DPSC stably silenced for p53. We observed that p53 knockdown is accompanied by p21 inhibition and induction of Bmi-1 expression (Fig. 5a). To verify these data, we exposed DPSC or SHED to MI-773, and observed that p53, MDM2, and p21 were upregulated while Bmi-1 was downregulated in a dose-dependent manner in both cell types (Fig. 5b). To further verify these data, we used another small molecule inhibitor of p53-MDM2 interaction (APG-115), and observed a dose-dependent increase in p53, p21, and MDM2 and a decrease in Bmi-1 expression (Fig. 5c). Then, we bypassed p53 and inhibited Bmi-1 with a small molecule inhibitor (PTC-209), and observed that although p53 expression remained unchanged, MDM2 and p21 expression were induced (Fig. 5d), suggesting the existence of a feedback loop leading to inverse and reciprocal regulation of p21 and Bmi-1. Notably, direct inhibition of Bmi-1 resulted in decreased expression of endothelial differentiation markers (i.e., VEGFR2, Tie-2) in pulp stem cells (Fig. 5e) and decreased the number of capillary-like sprouts in both DPSC and SHED cells in vitro (Fig. 5f, g; Supplementary Fig. S2).

### Ubiquitin/proteasome activity regulates Bmi-1 and vasculogenic differentiation of pulp stem cells

**Fig. 6:**
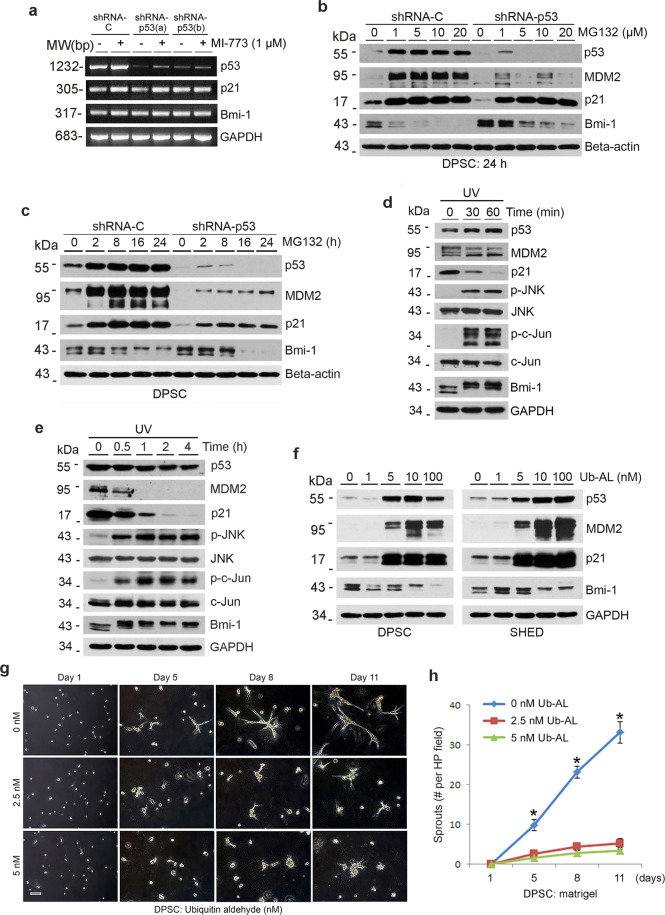
Ubiquitin/proteasome activity regulates Bmi-1 and vasculogenic differentiation of pulp stem cells. **a** shRNA-transduced DPSC were treated with 1 µM MI-773 for 24 h, RT-PCR for p53, p21, and Bmi-1. **b**, **c** shRNA-transduced DPSC were cultured with 0–20 µM proteasome inhibitor MG132 for 24 h (**b**), or 5 µM MG132 for indicated time points (**c**), western blots for p53, MDM2, p21, and Bmi-1. **d**, **e** SHED (**d**) and DPSC (**e**) were exposed to ultraviolet (UV) for 1 min, then cultured for time points, western blots were performed for p53, MDM2, p21, p-JNK, JNK, p-c-Jun, c-Jun, and Bmi-1. **f** DPSC and SHED were treated with 0–100 nM ubiquitin aldehyde for 24 h, western blots were performed for p53, MDM2, p21, and Bmi-1. **g**, **h** 1 × 10^4^ DPSC were seeded in growth factor-reduced matrigel and cultured with EGM2 in the presence of 0–2.5 nM ubiquitin aldehyde for different time points. **g** photographs of sprout in DPSC. Scale bar: 100 µm. **h** Graph depicting the number of sprouts formed in **g**. Asterisk indicates *p* < 0.001, as determined by one-way ANOVA followed by a post hoc test (Tukey’s test).

To understand how p53/p21 regulates Bmi-1 expression, we performed RT-PCR analyses with p53-silenced DPSC (or vector controls) exposed (or not) to MI-773. We observed that both, p21 and Bmi-1 are not regulated by p53 at the transcriptional level (Fig. 6a). Knowing that MDM2 binding promotes p53 degradation through the ubiquitin-dependent proteasome pathway [36] and that Bmi-1 is also reported to degrade through the ubiquitin-proteasome pathway [37], we exposed DPSC cells to the proteasome inhibitor MG132 for 24 hours (Fig. 6b, c). In vector control DPSC, we observed that inhibition of proteasome activity with MG132 is sufficient to induce p53, MDM2, and p21 expression, whereas mediating dose-dependent (Fig. 6b) and time-dependent (Fig. 6c) inhibition of Bmi-1 expression. Interestingly, these processes are independent of p53/MDM2 activity, as p53-silenced DPSC exposed to MG132 also showed dose-dependent (Fig. 6b) and time-dependent (Fig. 6c) induction of p21 and inhibition of Bmi-1 expression. To verify the inverse and reciprocal regulation of p21 and Bmi-1 using an alternate approach, we exposed DPSC to low-dose ultraviolet (UV) radiation for 1 minute and retrieved the cells after 30 min to 4 hours (Fig. 6d, e). It has been shown that low-dose UV-irradiation can inhibit p21 expression via a p53-independent mechanism [38–41]. Here, we observed that low-dose UV radiation caused p21 downregulation and activation of the JNK pathway (i.e., phosphorylation of JNK and c-Jun), which has been shown to regulate cell fate [42]. Interestingly, p21 downregulation was consistently accompanied by induction of Bmi-1 expression (Fig. 6d, e), corroborating results that we observed when shRNA-p21 was transduced into DPSC cells (Fig. 4a). And finally, these results were reproduced using a ubiquitin-deconjugation inhibitor (ubiquitin aldehyde) in both DPSC and SHED cells (Fig. 6f). These data suggest that p53, MDM2, and p21 are regulated through the ubiquitin-proteasome pathways, as the use of the ubiquitin aldehyde mediated a dose-dependent increase in expression levels. Here again, we observed an inverse and reciprocal regulation where an increase in p21 expression correlated with a decrease in Bmi-1 in DPSC. Notably, ubiquitin aldehyde treatment was sufficient to inhibit the vasculogenic differentiation of DPSC cells (Fig. 6g, h) and SHED cells in vitro without a visible effect on cell density (Supplementary Fig. S3).

### Regulation of p53 targets in DPSC

**Fig. 7:**
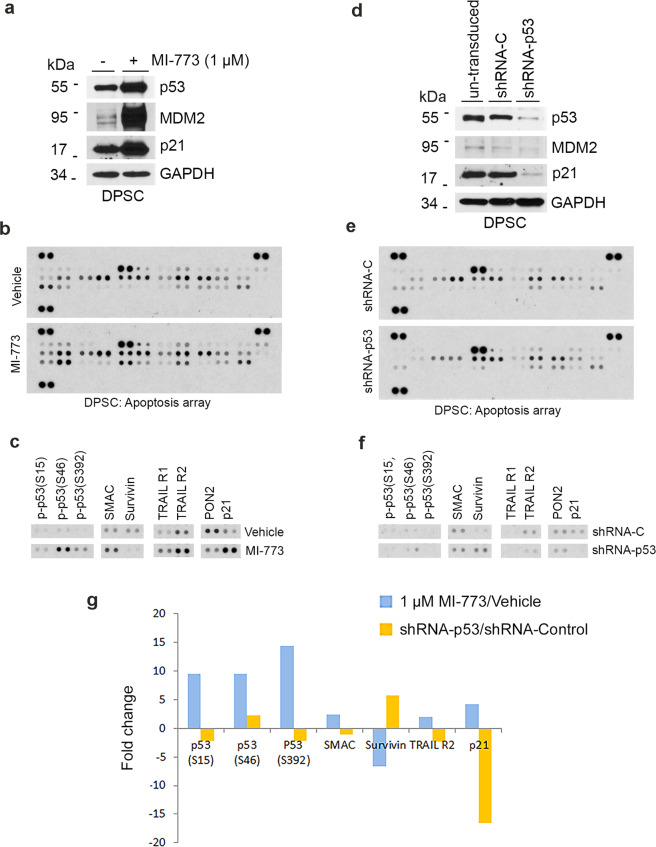
Expression of downstream targets of p53 in DPSC cells. **a** DPSC were treated with 1 µM MI-773 (or vehicle control) for 24 hours. Western blots for p53, MDM2, and p21 were used to verify the effect of MI-773 treatment on p53. **b**, **c** Protein arrays for evaluation of MI-773-treated DPSC versus control-treated DPSC. **c** Selected genes from **b** (full results are in Suppl Table 1). **d** DPSC were stably transduced with shRNA-control or shRNA-p53. Westerns blot for p53, MDM2, and p21 to verify the effect of shRNA constructs. **e**, **f** Protein array for evaluation of shRNA-p53 transduced DPSC versus shRNA-Control-transduced DPSC. **f** Selected genes from **e** (full results are in Suppl Table 2)**. g** Graph depicting the fold change in protein density from MI-773/vehicle from MI-773/vehicle **c** and from shRNA-p53/shRNA-control **f** showing inverse correlations in several p53 targets (e.g., p21, SMAC, and TRAILR2).

To understand the impact of p53 regulation in downstream targets in DPSC, we used MI-773 to stabilize MDM2 and upregulate p53 in DPSC, or shRNA-p53 to downregulate p53 in DPSC. As expected, p53, MDM2, and p21 were upregulated in MI-773-treated DPSC (Fig. 7a) and downregulated in shRNA-p53 transduced DPSC (Fig. 7d). Then we performed a human protein array and observed that MI-773 treatment induced upregulation of phosphor-p53-(S15, S46, S392), p21, tumor necrosis factor (TNF)-related apoptosis-inducing ligand-TRAIL receptor 2 (TRAILR2) [43], and second mitochondria-derived activator of caspase that participates in a feedback amplification loop to promote cytochrome c release and other mitochondrial events in apoptosis [44]. We also observed downregulation of the inhibitor of apoptosis survivin [45] (Fig. 7c, g, Supplementary Table 1). In contrast, knockdown of p53 induced expression of Survivin and inhibited expression of phosphor-p53 (S15, S392), p21, and TRAILR2 expression (Fig. 7f, g, Supplementary Table 2). In addition to inhibition of apoptosis, Survivin also plays important role in endothelial cells and smooth muscle cells in the regulation of angiogenesis [46] and vascular injury [47].

## Discussion

Temporal and spatial regulation of stem cell fate is a tightly regulated process that enables the use of stem cells in tissue regeneration and repair in response to specific environmental cues. In addition, the maintenance of a stem cell pool throughout the life of tissue requires self-renewal processes that are typically regulated via intercellular molecular crosstalk processes involving cells of the perivascular stem cell niche [9, 10, 48]. While most attention has been dedicated to processes that regulate stem cell differentiation, a less understood but equally important component of tissue homeostasis is mechanisms that maintain stemness by preventing stem cells from undergoing differentiation. Here, we unveiled the role of p53 as a gate-keeper for the differentiation of DPSC, with an emphasis on the vasculogenic fate of these cells. Although this work is focused on the study of primary human stem cells of dental origin, it is likely that these results are applicable to mesenchymal stem cells that reside in other tissues and organs. Importantly, although our work focuses on the impact of p53/p21 on the vasculogenic differentiation of pulp stem cells, our data do not exclude the possibility that similar signaling events regulate other differentiation pathways in these cells.

During development, endothelial cells arise from Flk1-expressing mesoderm cells [49], whereas mural cells are derived from mesoderm, neural crest, or epicardial cells [50–52]. Embryonic stem cells differentiate into endothelial cells upon signaling initiated by factors such as VEGF_165_, and into mural cells upon stimulation by factors such as PDGF-BB [53]. Mural cells assist endothelial cells in acquiring specialized functions that are required for blood vessel function and maturation [54]. We know that postnatal DPSC differentiate into functional blood vessels when transplanted into murine hosts [11, 12]. Using GFP-transduced pulp stem cells, we observed that while stem cells differentiate into vascular endothelial cells, the process of vessel maturation requires the recruitment of host mural cells. The process of vasculogenic differentiation of these stem cells is regulated by p53.

p53 is a tumor suppressor that functions through p21 to arrest cell proliferation and induce apoptosis [19, 55]. In addition, p53 has been implicated in processes that regulate the balance between stemness and differentiation during development [32]. We observed here that knockdown of p53 inhibited MDM2 and p21 expression, induced expression of Bmi-1, and vasculogenic differentiation of postnatal stem cells. In contrast, induction of p53, MDM2, and p21 with small molecule inhibitors of the p53-MDM2 binding resulted in decreased expression of Bmi-1 and inhibited vasculogenic differentiation. These data unveiled an intriguing inverse and reciprocal correlation between p53/p21 and Bmi-1 function in the regulation of the vasculogenic fate of DPSC.

The maintenance of a stem cell pool throughout the life of a tooth is important to maintain tissue homeostasis and to enable dental pulp tissue regeneration [48]. In physiological conditions, stem cells can differentiate and replace specialized cells that are dying (e.g., odontoblasts) to maintain the function of the pulp tissue over the lifetime of the tooth. Since odontoblasts are terminally differentiated, these cells are not capable of proliferating to generate daughter cells that could maintain the integrity of the odontoblastic layer. The replacement cells for these dead odontoblasts come from DPSC, which can be induced to undergo odontoblastic differentiation upon demand. In situations when the odontoblastic layer is partially lost (e.g., pulp exposure, deep caries), the replacement of lost odontoblasts also comes from undifferentiated cells that migrate from the center of the pulp tissue [56]. As such, stem cell self-renewal is critical for the long-term function and viability of the dental pulp tissue. Notably, we are beginning to understand the role of molecular crosstalk between cells of the perivascular niche (particularly endothelial cells) and stem cells that are mediated by IL-6/IL-6R [9] and SCF/c-Kit [10] on the self-renewal and long-term maintenance of the stem cell pool in the dental pulp tissue.

We have recently reported that only DPSC expressing VEGFR1 (10–20% of the total cell number) are capable of differentiating into endothelial cells, as VEGF signaling through VEGFR1 is required for induction of vasculogenic fate [57]. As such, even if all VEGFR1-expressing DPSCs differentiate into endothelial cells, the expression level of endothelial markers in DPSCs will be lower than the expression of the same markers in HDMEC, a pure population of endothelial cells.

Multiple lines of evidence support the concept that an inverse and reciprocal regulation of p21 and Bmi-1 is a necessary component of p53′s function in the regulation of vasculogenic differentiation, as follows: (A) direct knockdown of p21 is sufficient to induce Bmi-1 expression (independent of p53) and vasculogenic differentiation of DPSC; (B) direct inhibition of Bmi-1 with PTC-209 is sufficient to prevent vasculogenic differentiation of DPSC; (C) in p53 knockdown stem cells, the proteasome inhibitor MG132 induces dose and time-dependent increase in p21 expression coupled with inhibition of Bmi-1; (D) exposure to UV light for 1 minute inhibits p21 and induces Bmi-1 in DPSC; and (E), treatment with ubiquitin aldehyde stabilizes ubiquitin activity, induces p21 while inhibiting Bmi-1 and vasculogenic differentiation.

Collectively, these studies demonstrate that p53 triggers a series of intracellular events that involve an inverse and reciprocal regulation of p21 and Bmi-1 that defines the vasculogenic fate of DPSC. Surprisingly, knockdown of p53 is sufficient to induce vasculogenic differentiation of these cells. Considering that DPSC is multipotent, it is possible that the release of p53 “braking function” might be sufficient to allow the differentiation of these cells into other cell types. This is an exciting hypothesis that should be investigated in future studies. Within the limitations of this study, however, we conclude that p53 and p21 function as gatekeepers of the vasculogenic differentiation of DPSC.

## Materials and methods

### Cell culture

DPSC (PT-5025; Lonza, Walkersville, MD, USA) and SHED (provided by Songtao Shi, University of Pennsylvania) were cultured in α-minimum essential medium (Invitrogen, Carlsbad, CA, USA) supplemented with 5–20% fetal bovine serum (Invitrogen) and 1% penicillin/streptomycin (Invitrogen) at 37°C and 5% CO_2_. HDMEC (CC-2543; Lonza) were cultured in endothelial growth medium-2 for microvascular cells (EGM2-MV; CC-3202; Lonza). HUASMC (8030; ScienCell Research Laboratories; Carlsbad, CA, USA) were cultured in a smooth muscle cell medium with cell growth supplement (1101; ScienCell Research Laboratories). Mycoplasma-free cells were treated with 0–10 µM p53-MDM2 complex inhibitors, MI-773, or APG-115 (provided by Shaomeng Wang, University of Michigan) [34], 0–20 µM proteasome inhibitor MG132 (C2211; Millipore Sigma; Burlington, MA, USA) or 0–2.5 µM ubiquitin aldehyde, a potent and specific ubiquitin-deconjugation inhibitor (19-205; Upstate Cell Signaling Solution; Los Altos, CA, USA), 0–10 µM Bmi-1 inhibitor PTC-209 (5191; Tocris; Bristol, United Kingdom) for indicated time points.

### Western blots

Cells were lysed in 1% Nonidet P-40 (NP-40) lysis buffer (50 mM Tris-HCL, PH 7.4, 10% glycerol, 200 mM NaCl and 2 mM MgCl_2_) containing protease inhibitors. Protein lysates were loaded onto 8–15% sodium dodecyl sulphate–polyacrylamide gel electrophoresis (SDS–PAGE). Membranes were blocked with 5% non-fat milk in 1× TBS containing 0.3% Tween-20, then incubated with the following primary antibodies overnight at 4°C: rabbit anti-human VEGFR2 (SC-504), c-Jun (SC-1694); mouse anti-human VE-cadherin (SC-9989), CD31 (SC-365804), CD146 (SC-18837), MDM2 (SC-965), p53 (SC-126), phosphor-c-Jun (SC-822), phosphor-JNK (SC-6254), JNK (SC-1648), mouse anti-human β-actin conjugated with HRP (SC-47778HRP) (Santa Cruz Biotechnology; Santa Cruz, CA, USA); mouse anti-GAPDH (MAB374; Millipore Sigma); rabbit anti-human Tie-2 (7403), Bmi-1(6964), PDGFR-a (5241), PDGFR-β (4564), p21 (2947) (Cell Signaling; Danvers, MA, USA); mouse anti-human SMA-alpha; CBL171; Millipore Sigma). Affinity-purified secondary antibodies conjugated with horseradish peroxidase (Jackson Laboratories; West Grove, PA, USA) were used and immunoreactive proteins were visualized by SuperSignal West Pico chemiluminescent substrate (cat:34578; Thermo Scientific; Rockford, IL, USA). Here, and thereafter, in vitro experiments were performed three independent times to verify the reproducibility of the data.

### In vitro capillary-like sprouting assay

In all, 1 × 10^4^ DPSC, SHED, shRNA-control (scrambled sequence) or shRNA-p53, shRNA-p21 transduced DPSC and SHED were seeded in each well of 12-well plates (triplicate wells per experimental condition) coated with growth factor-reduced matrigel (354230, BD Biosciences; Bedford, MA, USA), and cultured with EGM2-MV medium with or without 0–0.5 µM MI-773, 0–5 nM ubiquitin aldehyde (Upstate Cell Signaling Solution), 0–2.5 µM PTC-209 (Tocris) for different time points. Capillary sprouts were counted in eight randomly selected microscopic fields (×100) in triplicate wells per condition and time point, as described [58]. Only connected capillary sprouts were counted, whereas single branches were not included. Here, and thereafter, the investigator was blinded for the experimental conditions at the time of data collection.

### Reverse transcriptase PCR

Total RNA was prepared in Trizol (Invitrogen) according to the manufacturer’s instructions. cDNA was synthesized with SuperScript II reverse transcriptase (RT) (11904-18; Invitrogen) and PCR was performed with Platinum Taq DNA Polymerase (10966-034; Invitrogen). The primers used in this study were, as follows: human p21, sense 5′-AGTCAGTTCCTTGTGGAGCC-3′, antisense 5′-GAAGGTAGAGCTTGGGCAGG-3′; human p53, sense 5′-GTGACACGCTTCCCTGGATTGG-3′, antisense 5′-GTCAGTCTGAGTCAGGCCCTTC-3′; human Bmi-1, sense 5′-CAGCGGTAACCACCAATCTT-3′, antisense 5′-AAAGTCTTGCCTGCTTTCCA-3′; human VEGFR1, sense 5′-ACTCCCTTGAACACGAGAGTTC-3′, antisense 5′-GATTTCTCA GTCGCAGGTAACC-3′; human VEGFR2 sense 5′-GCTGTCTCAGTGACAAACCCAT-3′, antisense 5′-CTCCCACATGGATTGGCAGAGG-3′; human CD146 sense 5′-GGAGCCAAACATCCAGGTCA-3′, antisense 5′-TTCATAGCCCCCACTGTGTT-3′; human CD31 sense 5′-TACTCAGTCATGGCCATGGT-3′, antisense 5′-TTGGCCTTGGCTTTCCTCAG-3′; human VE-cadherin sense 5′-CCTGGTATAACCTGACTGTG-3′, antisense 5′-TGTGATGGT GAGGATGCAGA-3′; human DSPP sense 5′-TCACAAGGGAGAAGGGAATG-3′, antisense 5′-TGCCATTTGCTGTGATGTTT-3′; human DMP-1 sense 5′-CAGGAGCACAGGAAAAGGAG-3′, antisense 5′-CTGGTGGTATCTTGGGCACT-3′; human GAPDH sense 5′-GACCCCTTCATTGACCTCAACT-3′, antisense 5′-CACCACCTTCTTGATGTCATC-3′. RT-PCR products were verified by electrophoresis in agarose gel.

### Flow cytometry

Surface markers of mesenchymal stem cells and endothelial cells were detected and analyzed by flow cytometry. The antibodies used for flow cytometry were, as follows: PE-conjugated rabbit anti-human VEGFR1 (bs-0170R-PE; Bioss Inc.; Woburn, MA, USA); FITC mouse anti-human CD146 (560846), APC mouse anti-human CD44 (559942), APC mouse anti-human CD73 (560847), APC mouse anti-human-CD90 (559869), APC mouse anti-human CD34 (560940) (BD Biosciences; San Jose, CA, USA) and APC mouse anti-human CD105 (323208) (Biolegend; San Diego, CA, USA); PE-rabbit IgG (bs-02950-PE; Bioss Inc.; Woburn, MA, USA), FITC mouse IgG (560126, 555748) and APC mouse IgG (555751) (BD Biosciences; San Jose, CA, USA) were used as isotype control.

### Immunohistochemistry and immunofluorescence

Four-µm-thick sections were deparaffinized and rehydrated. Antigen retrieval was performed with trypsin (Sigma) at 37°C for 30–60 min, and sections were incubated with rabbit anti-human CD31 (IHC-00055; Bethyl Laboratories; Montgomery, TX, USA), rabbit anti-factor VIII related antigen/Von Willebrand Factor Ab-1 (RB-281-A; Thermo Scientific; Waltham, MA, USA), mouse anti-human SMA-alpha (CBL171; Millipore Sigma) and rabbit anti-Turbo GFP (AB513; Evrogen; Moscow, Russia) overnight at 4^0^C. The MACH3 system (Biocare Medical) and Betazoid DAB (Biocare Medical; Pacheco, CA, USA) was utilized for visualization of immunohistochemistry (IHC) staining. Alexa Flour 488 goat-anti-rabbit IgG (green) (A11037; Life Technologies; Carlsbad, CA, USA) and Alexa Flour 594 goat-anti mouse IgG (red) (A11032; Life Technologies) were used as the secondary antibody to detect blood vessels labeled with anti-human-CD31, anti-Turbo GFP and anti-SMA-a primary antibody, respectively. Isotype-matched non-specific IgG (Jackson Laboratories; West Grove, PA, USA) was used as negative control.

### UV treatment

DPSC and SHED were exposed to UV light (254 nM, 150–200 mW/cm^2^) for 1 min, then cultured with a regular medium for the indicated time points. At the end of the experiment, cells were collected and Western blots were performed.

### p53 and p21 silencing

HEK293T cells were transiently co-transfected with the lentiviral packaging vectors psPAX2, pMD2G, and pLenti-GFP (kindly provided by Dr. Peter Friedl), shRNA-p53, shRNA-p21, or scramble sequence control (shRNA-C) (Vector Core, University of Michigan) by the calcium phosphate method. DPSC and SHED were infected with supernatants containing lentivirus and selected with 1 µg/ml puromycin (Sigma-Aldrich; St. Louis, MO, USA) for at least 1 week. Knockdown of p53 and p21 was verified by western blot. Stable transduction of GFP in the stem cells was confirmed by immunofluorescence microscopy.

### Human apoptosis array

In all, 200–400 µg protein lysates of shRNA-Control, shRNA-p53, MI-773-treated or vehicle-treated DPSC were used to evaluate p53 targets with human apoptosis array kit (ARY009, R&D systems; Minneapolis MN USA). Membranes were exposed to x-ray film for 2–10 min, the intensity of proteins was quantified by Image J software. The fold change of band density compared with controls was used to show the impact of treatment on protein expression.

### *SCID* mouse model of human DPSC-derived vasculogenesis

Human blood vessels derived from DPSC were generated in immunodeficient mice under a UCUCA approved protocol (PRO00009087), as described [33]. In brief, highly porous poly-l(lactic) acid (Boehringer Ingelheim; Ingelheim, Germany) scaffolds (*n* = 8 per experimental condition) measuring 6 mm × 6 mm × 1 mm were seeded with 10^6^ shRNA-control or shRNA-p53 transduced primary human DPSC (Lonza). Female, 6–8 week old SCID mice (CB.17.SCID; Taconic, Germantown, NY, USA) were anesthetized with ketamine and xylazine, and the biodegradable scaffolds containing cells were implanted bilaterally in the subcutaneous space of the dorsal region of each randomly assigned mouse. After 6 weeks, mice were euthanized, scaffolds were removed, fixed with 10% buffered formalin phosphate, and prepared for IHC and immunofluorescence staining. This study was repeated twice to verify the reproducibility of the data. The investigator was blinded for the experimental conditions.

### Statistical analysis

*t* test or one-way ANOVA followed by appropriate post hoc tests were performed using the SigmaStat 4.0 software (SPSS; Chicago, IL, USA). Graphs depict mean ± standard deviation throughout the manuscript. Sample sizes were for in vitro and in vivo studies were determined by power calculations using data published in previous publications (or pilot tests) as reference. The variance between groups was relatively similar in the studies included here. Statistical significance was determined at *p* < 0.05.

## Supplementary information

Supplemental Material

Suppl. Table 1

Suppl. Table 2

## Data Availability

The data that support the findings of this study are available from the corresponding author upon reasonable request.
