# Supplementary material for: Inverse and reciprocal regulation of p53/p21 and Bmi-1 modulates vasculogenic differentiation of dental pulp stem cells
Source: Cell Death Dis. 2021 Jun 24;12(7):644. doi: 10.1038/s41419-021-03925-z (PMC8225874; doi:10.1038/s41419-021-03925-z)
Supplement: Supplementary file 1 — Supplemental Material [file 41419_2021_3925_MOESM1_ESM.pdf]

# Supplementary Figure Legends

**Supplementary Figure S1.** MI-773 inhibits capillary sprout formation by SHED cells. **a** $1X10^4$ SHED were seeded in 12-well plates coated with growth factor reduced Matrigel and cultured in EGM2-MV with 0-0.5 µM MI-773 for 11 days. Photomicrographs of representative fields. Scale bar: 100 µm. **b** Graph depicting the numbers of sprout formed in (a). Asterisk indicates p<0.001, as determined by one-way ANOVA followed by a post-hoc test (Tukey's test).

**Supplementary Figure S2**. PTC-209 inhibits proliferation and capillary sprout formation of dental pulp stem cells. **a** 5 or 8 $X10^4$ DPSC or SHED (respectively) were seeded in each well of 6-well plates and cultured with standard medium in presence of 0 or 0.5 µM PTC-209 for up to 5 days. **b** DPSC were cultured with EGM2-MV in presence of 0-2.5 µM PTC-209 for up to 14 days. **c** Graph depicting the numbers of sprouting in (b). Three independent experiments were performed. Asterisk indicates p<0.001, as determined by one-way ANOVA followed by a post-hoc test (Tukey's test).

**Supplementary Figure S3**. Ubiquitin aldehyde prevents sprout formation of SHED. $1X10^4$ SHED were seeded in growth factor reduced Matrigel and cultured in EGM-2 with 0-5 nM ubiquitin aldehyde for 15 days. **a** Photographs of sprout of SHED. **b** Graph depicting the numbers of sprout formed in (a). Asterisk indicates p<0.001, as determined by one-way ANOVA followed by a post-hoc test (Tukey's test).

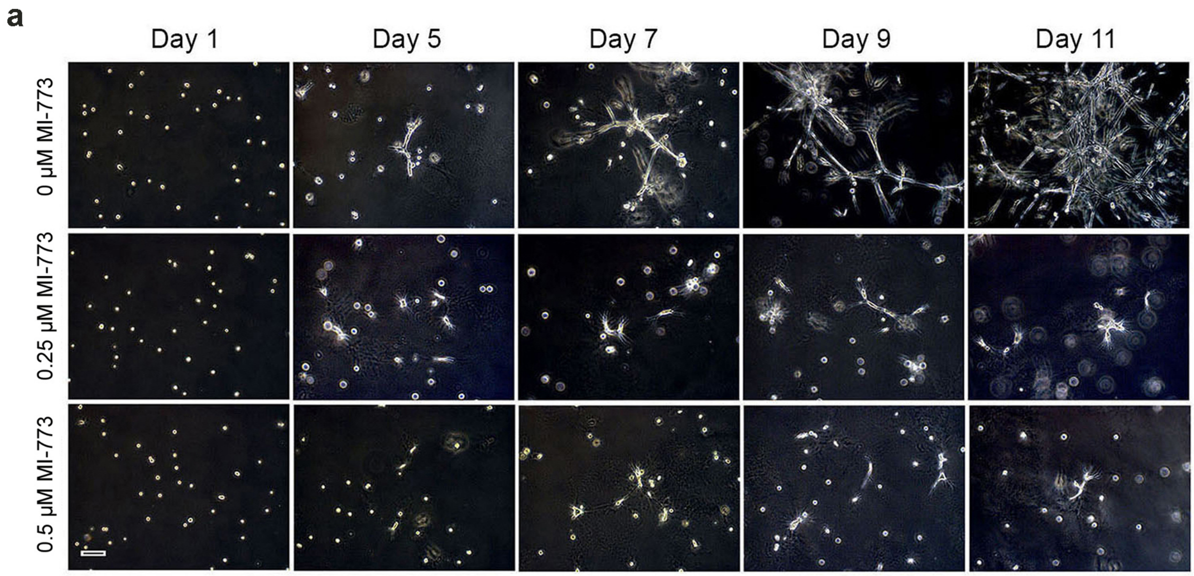


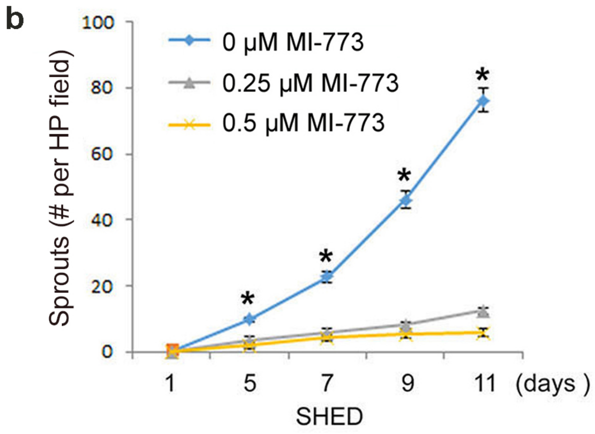


Supplementary Figure S1 - Zhang et al

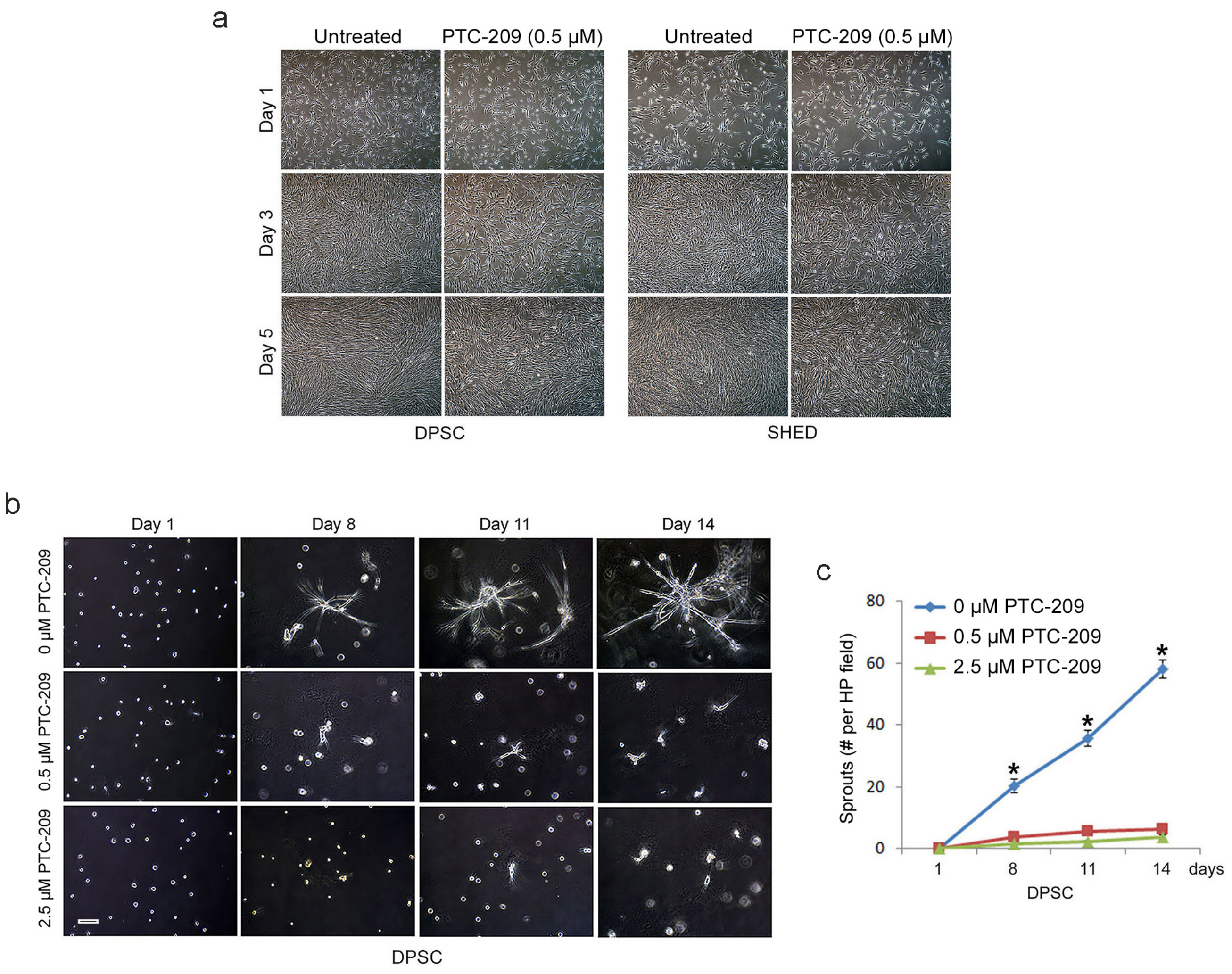


Supplementary Figure S2 - Zhang et al

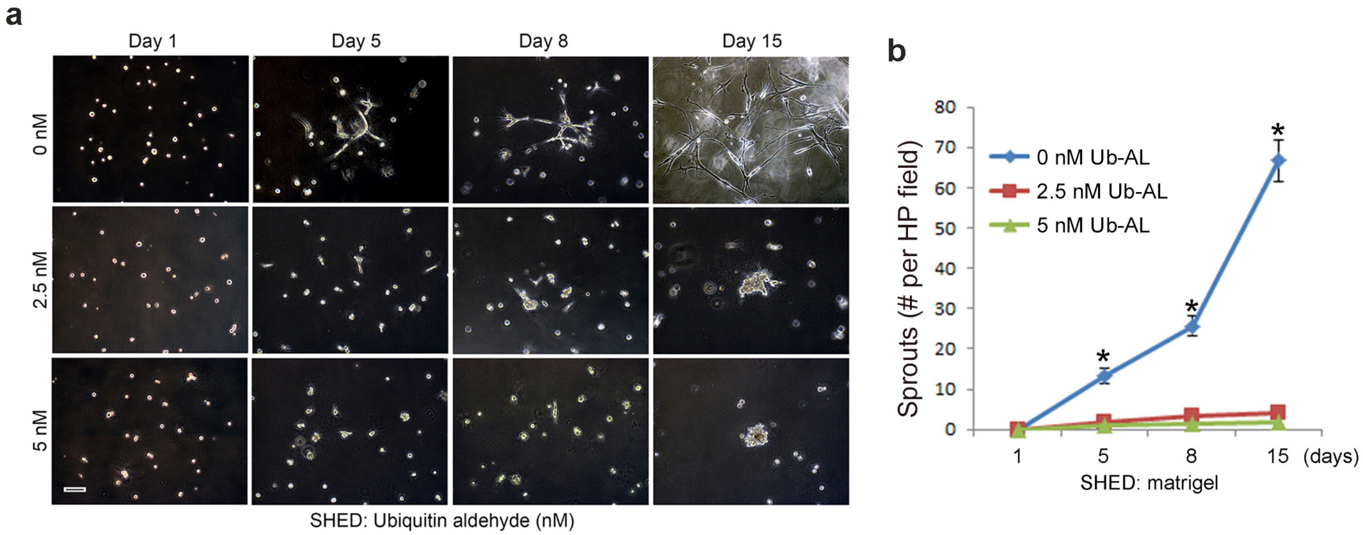


Supplementary Figure S3 - Zhang et al
