# Supplementary material for: Inverse and reciprocal regulation of p53/p21 and Bmi-1 modulates vasculogenic differentiation of dental pulp stem cells
Source: Cell Death Dis. 2021 Jun 24;12(7):644. doi: 10.1038/s41419-021-03925-z (PMC8225874; doi:10.1038/s41419-021-03925-z)
Supplement: Supplementary file 2 — Suppl. Table 1 [file 41419_2021_3925_MOESM2_ESM.docx]

**Supplementary Table 1.** Table containing the raw data derived from human apoptosis protein array of MI-773-treated DPSC compared to vehicle-treated controls. DPSC were treated with 1 µM MI-773 (or vehicle) for 24 hours. Protein lysates were collected and the protein array was performed. Membranes were exposed to x-ray films. Band densities were normalized against controls and quantified. Table depicts the fold change of band density as compared with controls.

| ***Protein*** | ***Vehicle control*** | ***1 µM MI-773*** | ***Fold change*** |
| --- | --- | --- | --- |
| B1, B2 Bad | 642.163 | 1352.548 | + 2,11 |
| B3, B4 Bax | 860.991 | 2490.790 | + 2.89 |
| B5, B6 Bcl-2 | NA | NA |  |
| B7, B8 Bcl-x | NA | NA |  |
| B9, B10 Pro-Caspase-3 | 12695.782 | 13985.368 | + 1.1 |
| B11, B12 Cleaved-Caspase-3 | 1672.841 | 2449.841 | + 1.46 |
| B13, B14 Catalase | 379.849 | 665.335 | + 1.75 |
| B15, B16 cIAP-1 | 1966.134 | 2389.426 | + 1.22 |
| B17, B18 cIAP-2 | 538.506 | 418.263 | - 1.29 |
| B19, B20 Claspin | 1016.234 | 364.021 | - 2.79 |
| B21, B22 Clusterin | 374.263 | 463.678 | + 1.24 |
| B23, B24 Cytochrome C | 901.698 | 1486.184 | + 1.65 |
| C1, C2 TRAIL R1 | 1478.891 | 4056.033 | + 2.74 |
| C3, C4 TRAIL R2 | 4860.033 | 9695.225 | + 1.99 |
| C5, C6 FADD | 5090.154 | 5922.397 | + 1.17 |
| C7, C8 Fas/TNFRSF6/CD95 | 11179.004 | 12934.296 | + 1.16 |
| C9, C10 HIF-1α | 6694.518 | 7782.690 | + 1.16 |
| C11, C12 HO-1/HMOX1/HSP32 | 7476.104 | 9512.518 | + 1.27 |
| C13, C14 HO-2/HMOX2 | 2976.376 | 4030.619 | + 1.35 |
| C15, C16 HSP27 | 9472.376 | 9390.690 | - 1.01 |
| C17, C18 HSP60 | 8419.225 | 8784.861 | + 1.04 |
| C19, C20 HSP70 | 3836.619 | 4242.912 | + 1.11 |
| C21, C22 HTRA2/Omi | 1848.255 | 3200.033 | + 1.73 |
| C23, C24 Livin | 169.192 | 602.163 | + 3.36 |
| D1, D2 PON2 | 7202.447 | 3949.154 | - 1.82 |
| D3, D4 p21/CIP/CDKN1A | 2716.497 | 11438.882 | + 4.21 |
| D5, D6 p27/Kip1 | 222.849 | 726.335 | + 3.26 |
| D7, D8 Phospho-p53(S15) | 625.749 | 1196.648 | + 9.52 |
| D9, D10 Phospho-p53(S46) | 828.991 | 7942.861 | + 9.58 |
| D11, D12 Phospho-p53(S392) | 167.607 | 2417.962 | + 14.43 |
| D13, D14 Phospho-Rad17(S653) | 698.991 | 1510.255 | + 2.16 |
| D15, D16 SMAC/Diablo | 2258.962 | 5531.569 | + 2.45 |
| D17, D18 Survivin | 2394.962 | 362.678 | - 6.60 |
| D19, D20 TNF-R1/TNFRSF1A | 2036.184 | 3191.497 | + 1.57 |
| D21, D22 XIAP | 6649.569 | 9491.933 | + 1.42 |
| D23, D24 PBS (Negative Control) |  |  |  |

**Note:** Reference spots, A1, A2, A23, A24, E1, E2
