# Supplementary material for: Inverse and reciprocal regulation of p53/p21 and Bmi-1 modulates vasculogenic differentiation of dental pulp stem cells
Source: Cell Death Dis. 2021 Jun 24;12(7):644. doi: 10.1038/s41419-021-03925-z (PMC8225874; doi:10.1038/s41419-021-03925-z)
Supplement: Supplementary file 3 — Suppl. Table 2 [file 41419_2021_3925_MOESM3_ESM.docx]

**Supplementary Table 2.** Table containing the raw data derived from human apoptosis protein array of shRNA-p53 transduced DPSC compared to shRNA-Control. Protein lysates were collected and the protein array was performed. Membranes were exposed to x-ray films. Band densities were normalized against controls and quantified. Table depicts the fold change of band density as compared with controls.

| ***Protein*** | ***shRNA-Control*** | ***shRNA-p53*** | ***Fold change*** |
| --- | --- | --- | --- |
| B1, B2 Bad | 403.849 | 113.364 | - 3.56 |
| B3, B4 Bax | 226.607 | 289.021 | + 1.28 |
| B5, B6 Bcl-2 | NA | NA |  |
| B5, B6 Bcl-x | NA | NA |  |
| B9, B10 Pro-Caspase-3 | 14156.246 | 14520.095 | + 1.03 |
| B11, B12 Cleaved –Caspase-3 | 651.577 | 996.062 | + 1.53 |
| B13, B14 Catalase | 282.192 | 247.435 | - 1.14 |
| B15, B16 cIAP-1 | 1033.820 | 1083.355 | + 1.05 |
| B17, B18 cIAP-2 | 235.607 | 49.536 | - 4.76 |
| B19, B20 Claspin | 145.778 | 792.820 | + 5.44 |
| B21, B22 Clusterin | 264.021 | 233.192 | - 1.13 |
| B23, B24 Cytochrome C | 322.435 | 295.021 | - 1.09 |
| C1, C2 TRAIL R1 | NA | NA |  |
| C3, C4 TRAIL R2 | 2040.548 | 865.870 | - 2.36 |
| C5, C6 FADD | 3678.255 | 3659.447 | - 1.01 |
| C7, C8 Fas/TNFRSF6/CD95 | 10220.740 | 3799.154 | - 2.69 |
| C9, C10 HIF-1α | 4896.497 | 8357.610 | + 1.71 |
| C11, C12 HO-1/HMOX1/HSP32 | 8662.983 | 9697.397 | + 1.12 |
| C13, C14 HO-2/HMOX2 | 1023.648 | 1496.790 | + 1.46 |
| C15, C16 HSP27 | 8612.569 | 8374.276 | - 1.03 |
| C17, C18 HSP60 | 7911.033 | 8477.569 | + 1.07 |
| C19, C20 HSP70 | 2557.376 | 3275.669 | + 1.28 |
| C21, C22 HTRA2/Omi | 802.406 | 1351.548 | + 1.68 |
| C23, C24 Livin | NA | NA |  |
| D1, D2 PON2 | 2408.790 | 1777.477 | - 1.36 |
| D3, D4 p21/CIP/CDKN1A | 1639.598 | 99.192 | - 16.53 |
| D5, D6 p27/Kip1 | NA | NA |  |
| D7, D8 Phospho-p53(S15) | 1505.962 | 697.113 | - 2.16 |
| D9, D10 Phospho-p53(S46) | 1311.012 | 2892.619 | + 2.21 |
| D11, D12 Phospho-p53(S392) | 497.820 | 234.849 | - 2.12 |
| D13, D14 Phospho-Rad17(S653) | 601.820 | 761.820 | + 1.17 |
| D15, D16 SMAC/Diablo | 2740.083 | 2728.669 | - 1.004 |
| D17, D18 Survivin | 628.749 | 3594.326 | + 5.72 |
| D19, D20 TNF-R1/TNFRSF1A | 1251.255 | 1591.719 | + 1.27 |
| D21, D22 XIAP | 8078.225 | 9334.933 | + 1.16 |
| D23, D24 PBS (Negative Control) |  |  |  |

**Note:** Reference spots, A1, A2, A23, A24, E1, E2
